# Optimizing Bi-LSTM networks for improved lung cancer detection accuracy

**DOI:** 10.1371/journal.pone.0316136

**Published:** 2025-02-24

**Authors:** Su Diao, Yajie Wan, Danyi Huang, Shijia Huang, Touseef Sadiq, Mohammad Shahbaz Khan, Lal Hussain, Badr S. Alkahtani, Tehseen Mazhar

**Affiliations:** 1 Department of Industrial & Systems Engineering, Auburn University, Auburn, Alabama, United States of America; 2 Department of Computer Science, Brown University, Providence, RI, United States of America; 3 Department of Chemical Engineering, Columbia University, New York City, NY, United States of America; 4 Fu Foundation School of Engineering and Applied Science, Fu Foundation School of Engineering and Applied Science, Columbia University, New York, NY, United States of America; 5 Department of Information and Communication Technology, Centre for Artificial Intelligence Research (CAIR), University of Agder, Grimstad, Norway; 6 Children’s National Hospital, Washington, DC, United States of America; 7 Department of Computer Science and Information Technology, The University of Azad Jammu and Kashmir, Chattar Kalas Campus, Muzaffarabad, Pakistan; 8 Department of Computer Science, Neelum Campus, The University of Azad Jammu and Kashmir, Azad Kashmir, Pakistan; 9 Department of Mathematics, King Saud University, Riyadh, Saudi Arabia; 10 School of Computer Science, National College of Business Administration and Economics, Lahore, Pakistan; 11 Department of Computer Science and Information Technology, School Education Department, Government of Punjab, Layyah, Pakistan; Istanbul Sabahattin Zaim University: Istanbul Sabahattin Zaim Universitesi, TÜRKIYE

## Abstract

Lung cancer remains a leading cause of cancer-related deaths worldwide, with low survival rates often attributed to late-stage diagnosis. To address this critical health challenge, researchers have developed computer-aided diagnosis (CAD) systems that rely on feature extraction from medical images. However, accurately identifying the most informative image features for lung cancer detection remains a significant challenge. This study aimed to compare the effectiveness of both hand-crafted and deep learning-based approaches for lung cancer diagnosis. We employed traditional hand-crafted features, such as Gray Level Co-occurrence Matrix (GLCM) features, in conjunction with traditional machine learning algorithms. To explore the potential of deep learning, we also optimized and implemented a Bidirectional Long Short-Term Memory (Bi-LSTM) network for lung cancer detection. The results revealed that the highest performance using hand-crafted features was achieved by extracting GLCM features and utilizing Support Vector Machine (SVM) with different kernels, reaching an accuracy of 99.78% and an AUC of 0.999. However, the deep learning Bi-LSTM network surpassed both methods, achieving an accuracy of 99.89% and an AUC of 1.0000. These findings suggest that the proposed methodology, combining hand-crafted features and deep learning, holds significant promise for enhancing early lung cancer detection and ultimately improving diagnosis systems.

## 1. Introduction

Lung cancer remains the most commonly diagnosed cancer and the leading cause of cancer death globally, particularly among men. According to recent estimates of 2024, approximately 2.5 million cases of lung cancer occur worldwide each year, accounting for roughly one in eight cancers. Tragically, 1.8 million deaths are attributed to lung cancer annually, representing one in five cancer-related deaths with non-small cell lung cancer (NSCLC) constituting roughly 85% of these diagnoses [1, 2]. Early detection of NSCLC is crucial for improved outcomes. Two primary treatment approaches for NSCLC include radiofrequency ablation and stereotactic body radiotherapy (SBRT).

Lung cancer is primarily categorized into two main types: small cell lung carcinoma (SCLC) and non-small cell lung carcinoma (NSCLC). These subtypes differ significantly in their biological behavior, including growth patterns, metastatic potential, and response to treatment. While NSCLC generally progresses more slowly, SCLC is highly aggressive, forming tumors rapidly and spreading to other parts of the body. Cigarette smoking is a well-established primary risk factor for lung cancer, with the cumulative exposure directly correlated to disease risk [3]. Lung cancer remains a leading global cause of cancer-related deaths, claiming over 1.4 million lives annually [4, 5]. Accurate and timely diagnosis is crucial for effective lung cancer treatment. Pathologists play a vital role in this process by carefully examining microscopic tissue samples, known as histopathology slides, to confirm the presence of lung cancer and determine its specific type and subtype [6, 7]. Adenocarcinoma and squamous cell carcinoma are the two primary subtypes of non-small cell lung cancer, distinguished through meticulous pathological analysis [8, 9].

SCLC is an aggressive form of lung cancer strongly linked to cigarette smoking. Its development is influenced by genetic factors such as proto-oncogenes, autocrine growth loops, and tumor-suppressor genes. These biological differences result in distinct treatment approaches and prognoses for SCLC compared to NSCLC. Early detection is crucial for improving NSCLC survival rates, which can range from 35% to 85% depending on tumor stage and type [10–12]. Unfortunately, late diagnosis remains common, leading to a five-year survival rate of only 16% for NSCLC. While initial chemotherapy responses for SCLC exceed 60%, the disease’s rapid recurrence results in a dismal five-year survival rate of 6%, with little improvement over the past two decades. Cigarette smoking is the primary risk factor for approximately 90% of lung cancer cases. However, other factors, including exposure to air pollution, radon, asbestos, and chronic infections, also contribute to the development of lung cancer. Additionally, genetic predisposition plays a role, with both inherited and acquired genetic alterations influencing susceptibility to the disease. Lung cancer treatment options are diverse and include surgery, radiation therapy, chemotherapy, and targeted therapies. The optimal treatment approach is determined by individual patient characteristics and disease stage [13].

The discovery of X-rays and radiation in the late 19th century revolutionized medical imaging, enabling non-invasive visualization of internal structures and paving the way for radiation-based cancer treatments. By the 1960s, the collaborative efforts of radiologists and surgeons, combined with advancements in computer technology, led to the accumulation of substantial cancer data. In the subsequent decades, medical imaging has undergone significant advancements. Computed tomography (CT) scans provide detailed cross-sectional images of the lungs and tumors, making them invaluable for treatment planning. Chest CT scans are crucial for staging lung cancer, while abdominal CT scans aid in detecting metastatic disease [14]. Positron emission tomography (PET) scans utilize a radioactive tracer to visualize metabolically active tissues, such as cancer cells. This modality is indispensable for assessing tumor spread to lymph nodes and distant organs [15]. Magnetic resonance imaging (MRI) excels in visualizing soft tissues, making it particularly useful for evaluating brain metastases in lung cancer patients [16]. These imaging modalities have become essential tools in the diagnosis and management of lung cancer, providing crucial information for treatment decisions and prognosis.

## 2. Literature background

The advent of powerful computing systems has accelerated the development of deep learning (DL), a subset of artificial intelligence (AI) that employs complex neural networks. Convolutional neural networks (CNNs), a cornerstone of D, have surpassed traditional neural networks by utilizing significantly deeper architectures [17]. This architecture has enabled remarkable achievements in various domains, including image recognition and segmentation [18, 19], big data analysis [20, 21], speech recognition [22], and complex tasks such as machine translation, autonomous vehicles, and agricultural disease detection [23, 24]. Inspired by the human brain’s structure, CNNs consist of interconnected layers of neurons. These models are trained using supervised learning techniques. In this study, we leveraged transfer learning, applying pre-trained CNN models to a new dataset of lung cancer images. Effective feature extraction and data preparation are essential for optimal CNN performance. To this end, we extracted traditional hand-crafted features like GLCM and Haralick textures from lung cancer (NSCLC and SCLC) patient data. These features were optimized and subsequently employed with machine learning algorithms, including Naive Bayes, decision trees, and SVM with various kernels. To explore the potential of deep learning further, we implemented LSTM networks for lung cancer detection.

The discovery of radiation and X-rays in the late 19th century revolutionized medical imaging, enabling physicians to examine the human body and explore non-surgical cancer treatments. Collaborative efforts between radiologists and surgeons, coupled with the advent of computers in 1968, led to the accumulation of significant cancer data. Over the past five decades, substantial research has been dedicated to this field. To assess the stage of lung cancer, various imaging modalities are employed. Computed Tomography (CT) scans, which provide detailed images of the anatomy and lung tumors, are crucial for treatment planning [25]. Chest CT scans are essential for cancer staging, while abdominal CT scans are used to identify secondary tumors and metastases. Positron Emission Tomography (PET) scans, which utilize radioactive sugar to detect rapidly metabolizing cancer cells, are invaluable for identifying the spread of cancer to lymph nodes or other organs [26]. Magnetic Resonance Imaging (MRI) scans are another valuable tool, particularly for examining the brain. Brain MRI scans can help determine the extent of tumor spread within the brain [16].

X-rays provide valuable insights into the functional and structural aspects of the human body. However, the radiation exposure associated with CT scans can compromise image quality. To address this, researchers have explored machine learning (ML) techniques to improve the analysis and interpretation of medical images. ML techniques have demonstrated significant potential in enhancing the accuracy and precision of lung cancer prognosis and prediction, often surpassing traditional expert-based systems and statistical methods [27]. Computer-aided diagnosis (CAD) systems have emerged as powerful tools for characterizing and identifying various lung lesions. These systems can directly extract relevant features from X-ray images, enabling the classification of lung tumors as benign or malignant [28]. Several imaging modalities are employed to assess the stage of lung cancer. CT scans of the chest and abdomen provide detailed images of lung tumors and anatomy, aiding in treatment planning. Chest CT scans are crucial for cancer staging, while abdominal CT scans help identify secondary tumors and metastases. PET scans, which utilize radioactive sugar to detect rapidly metabolizing cancer cells, are useful for identifying the spread of cancer to lymph nodes or other organs.

Recent advancements in machine learning have led to significant applications in medical diagnostics, including lung disease prediction. To assess feature importance, researchers have employed various standard toolkits [29]. Additionally, machine learning algorithms have been developed to predict the need for CT exams in emergency departments (EDs), aiding in resource allocation and patient flow management [30]. Electronic nose (e-nose) technology has emerged as a promising tool for distinguishing between healthy individuals and those with chronic obstructive pulmonary disease (COPD) by analyzing volatile organic compounds in exhaled breath [31, 32]. Liquid biopsy, which involves analyzing circulating biomarkers such as cell-free DNA, tumor cells, and microRNAs, has shown potential for diagnosing and detecting lung cancer [33]. Combining multiple biomarkers with computational tools can further enhance the accuracy of lung cancer diagnosis and prognosis. Blood cadmium levels have been identified as a potential biomarker for lung cancer, particularly in former smokers [34]. Machine learning techniques, combined with feature extraction and selection, have been applied to improve the accuracy of lung cancer detection in electronic health records, leading to enhanced diagnosis and treatment [35–37]. Researchers have explored the use of machine learning techniques in conjunction with the Internet of Things (IoT) to improve lung cancer prediction [38], E-nose systems have also been developed to analyze exhaled breath and classify patients based on their respiratory health status, including COPD, lung cancer, and asthma [38, 39]. Machine learning has a long history of application in medical research. For instance, it has been used to analyze brain levels of polyamines and histamine in individuals exposed to extreme conditions [40].

Chaunzwa et al. developed a supervised CNN predictor based on the VGG network to forecast early-stage ADC and SCC in lung cancer patients [41]. While validated using real-time data from Massachusetts General Hospital, the model achieved a modest 71% AUC, likely due to limitations in preprocessing and image segmentation. Chaturvedi et al. conducted a comprehensive review of contemporary lung cancer detection and classification strategies [42]. Recent approaches commonly employ supervised learning algorithms, such as SVM, KNN, and CNN, often utilizing standard datasets like LIDCIDRI, LUNA 16, and Super Bowl Dataset 2016 [45]. Masood et al. developed a computer-aided prognosis (CAD) system using a 3D-DCNN approach, trained and validated on LUNA16, ANODE09, and LIDC-IDRI datasets [43]. El Nabi et al. proposed a novel deep learning method combining binary particle swarm optimization with a decision tree (BPSO-DT) and a convolutional neural network to classify different cancer types based on tumor RNA sequences [44]. The study evaluated performance metrics such as recall, precision, and F1-score. Abdulghani and Al Ahmad explored label-free approaches for cell categorization, utilizing optical profiles and Prony methodologies [51]. He et al. identified signature genes by enhancing tobacco exposure pattern (TEP) classification models and analyzing their interconnections at various biological stages. TTZ, a novel technique for separating core properties, was integrated into the TEP classification model as an input parameter [45]. Using two independent LUAD datasets, 34 genes were identified as tobacco-related mutation classic genes. The TEP classification model achieved a precision of 94.65% for the training dataset and 91.85% for the testing dataset.

The medical imaging problem requires the better quality images for improved analysis. We first improved the quality of images using robust image enhancement techniques. The machine learning algorithms requires the static features extraction relevant to the problem. Moreover, the performance of machine learning and deep learning LSTM models requires to the hyperparameters optimization. Recent studies utilized fewer texture, statistical and morphological features on this dataset to train the machine learning classifiers. Moreover, the traditional machine learning algorithms with default parameters were utilized. In this study, we first extracted the most robust GLCM based features to capture the more relevant information from lung cancer imaging types to properly distinguish these types. The CNN models are computationally complex and unable to implement on edge devices. We thus, optimized the parameters of machine learning and deep learning LSTM algorithms using Bayesian optimization a more powerful parameters optimization method than random and grid search method. We also conducted a computational performance analysis. The results demonstrate that our proposed approach achieved the highest detection performance.

## 3. Materials and methods

### 3.1. Dataset

The dataset for this study was obtained from a publicly accessible web-based repository maintained by LCA the sole national non-DICOM format images from 76 patients utilized in our previous studies in [46–48]. Of these, 568 images belong to SCLC subjects, while 377 images represent NSCLC subjects from CT images.

### 3.2. Pre-processing

Following image pre-processing methods were utilized on lung cancer images.

#### 3.2.1. Image resize

We used, "inter area" is a type of interpolation that is used to resize images in a way that produces smooth, accurate results. In computer vision, interpolation is a method of estimating the value of a pixel in an image based on the values of surrounding pixels [49]. The "inter area" option specifies that the interpolation should be performed using the area-based method. In the area-based method, the value of the pixel is calculated based on the average value of the pixels in the area surrounding it. This method is typically used for resizing images, where the goal is to reduce the size of the image by reducing the number of pixels. Because the area-based method considers the values of multiple pixels, it can produce smoother, more accurate results than other interpolation methods.

#### 3.2.2. Data augmentation

Data augmentation is a method of creating additional data samples from existing ones in order to artificially increase the size of a dataset [50]. This can be useful when training machine learning models, especially when the available dataset is small or not representative of the problem being addressed. There are various techniques for data augmentation, including adding noise to the data, applying transformations to the data, and generating synthetic data by combining or modifying existing samples. Data augmentation can improve the generalization of a model by introducing variations in the training data that the model may encounter in the real world and can also help prevent overfitting.

### 3.3. Hand-crafted features extraction

This study commenced by extracting hand-crafted features from lung cancer images. Specifically, GLCM features were computed, as detailed in references [25–30]. Subsequently, supervised machine learning algorithms were applied for classification. These algorithms included Naive Bayes, decision trees, and SVM with diverse kernels. Comprehensive details of these algorithms can be found in references [51–56].

### 3.4. Deep learning Long Short-Term Memory (LSTM)

**Fig 1:**
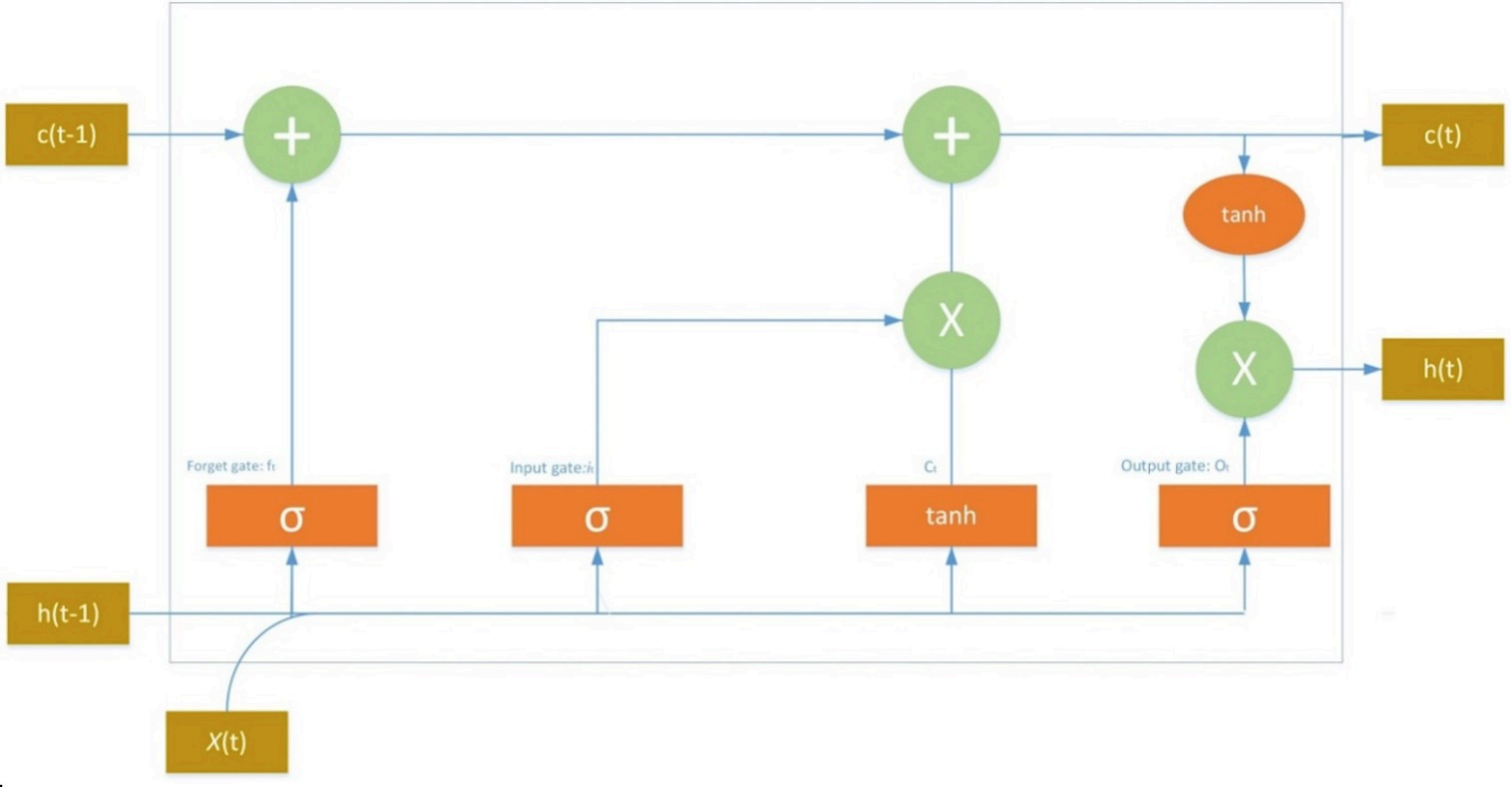
LSTM architecture gates: σ: Sigmoid activation function. tanh: Hyperbolic tangent activation function, Operations: +: Addition, ×: Multiplication, States: X(t): input state, c(t): Current cell state, c(t-1): previous cel state, h(t): Current hidden state, h(t-1): previous hidden state.

The LSTM networks, introduced by Hochreiter et al. [57] have become a prominent architecture within RNNs Particularly adept at handling time series data, LSTMs excel at capturing dependencies across both short and long temporal scales [58]. Unlike traditional RNNs, which suffer from the vanishing gradient problem [59], LSTMs mitigate this issue through the introduction of a cell state—a mechanism for preserving information over extended periods. This cell state, often visualized as a horizontal line in diagrams [60], is regulated by input, forget, and output gates. These gates regulate the flow of information into, out of, and within the cell state, enabling LSTMs to effectively capture long-term dependencies in sequential data. [57, 61]. Consequently, LSTMs are widely adopted for time series forecasting due to their capacity to capture both short-term and long-term patterns. Fig 1 illustrates the LSTM architecture.

**Memory cell’s role:** "The memory cell, considered LSTM’s core innovation, stores past information relevant to the current state."

**Forget gate:** "The forget state *f*_*t*_ first identifies irrelevant information from the previous state. A sigmoid function is then applied to these values, essentially determining how much of this information to forget (values closer to 0 are forgotten, while values closer to 1 are retained).


$$f_{t}=\sigma \left(W_{f}⬝\left[h_{t-1},x_{t}\right]+b_{f}\right)$$ (1)

The second step focuses on identifying which new data is crucial to store in the cell state. An additional sigmoid layer, called the "input gate layer," determines the importance of the current input. This information is then combined with the forget gate’s output to control how much new data gets added to the cell. Then, a *tanh* function is used to create a vector ${\tilde{c}}_{t}$ of novel values that ought to be updated embarked upcoming state.


$$i_{t}=\sigma \left(W_{i}⬝\left[h_{t-1},x_{t}\right]+b_{i}\right)$$ (2)

$${\tilde{c}}_{t}=tanh\left(W_{c}⬝\left[h_{t-1},x_{t}\right]+b_{c}\right)$$ (3)

The new candidate values for updating are represented by

$$c_{t}=f_{t}\;*\;c_{t-1}+i_{t}\;*\;{\tilde{c}}_{t}$$ (4)

The final step determines the output of the cell. This step involves two parts:

1. A sigmoid function acts as an output gate, filtering the cell state information. This ensures only relevant information contributes to the output.
2. The filtered cell state is then multiplied by the output of another function (potentially a tanh function) to create the final output value.

$$o_{t}=\sigma \left(W_{o}⬝\left[h_{t-1},x_{t}\right]+b_{o}\right)$$ (5)

$$h_{t}=o_{t}\;*\;\tanh\left(c_{t}\right)$$ (6)

In Eqs (1–5), *W*_*i*_, *W*_*f*_, *W*_*c*_, *W*_*o*_ shows how the weight matrices and bias vectors are represented by *b*_*i*_, *b*_*f*_, *b*_*c*_, *b*_*o*_ vectors.

Our LSTM model comprised a single layer with 48 neurons and employed the ReLU activation function [62, 63]. Hyperparameters were optimized to maximize model performance as reflected in the section 3.7.

### 3.5. Bi-LSTM

Long Short-Term Memory (LSTM) networks are an extension of Recurrent Neural Networks (RNNs) designed to address the vanishing and exploding gradient problems inherent in traditional RNNs. LSTMs employ a sophisticated architecture with three gates (input, forget, and output) and a cell memory state. This structure, illustrated in Fig 3, enables LSTMs to effectively capture long-term dependencies in sequential data. The mathematical expressions governing the behavior of these gates and the cell memory state are as follows

$$F_{g}=\sigma \left(W_{F}⬝h_{t-1}+R_{F}K_{d}+B_{F}\right)$$ (7)

$$I_{g}=\sigma \left(W_{I}⬝h_{t-1}+R_{I}K_{d}+B_{I}\right)$$ (8)

$$O_{g}=\sigma \left(W_{O}⬝h_{t-1}+R_{O}K_{d}+B_{o}\right)$$ (9)

$$C_{g}=F_{g}\;*\;C_{g-1}+I_{g}\;*\;\tanh\left(W_{C}⬝h_{t-1}+R_{C}K_{d}+B_{C}\right)$$ (10)

Here, *W*_*F*_, *W*_*I*_, *W*_*O*_, *W*_*C*_ and *B*_*F*_, *B*_*I*_, *B*_*O*_, *B*_*C*_ represent the weight matrices and biases for the forget gate, input gate, output gate, and cell memory state, respectively. The σ symbol denotes the sigmoid function, and * represents element-wise multiplication. Cg represents the cell memory gate, and *h*_*t*-1_ represents the previous hidden vector. *R*_*F*_, *R*_*I*_, *R*_*O*_, *R*_*C*_ denote the correlation coefficients, and *K*_*d*_ represents the k-space data fed to the LSTM layer.

**Fig 2:**
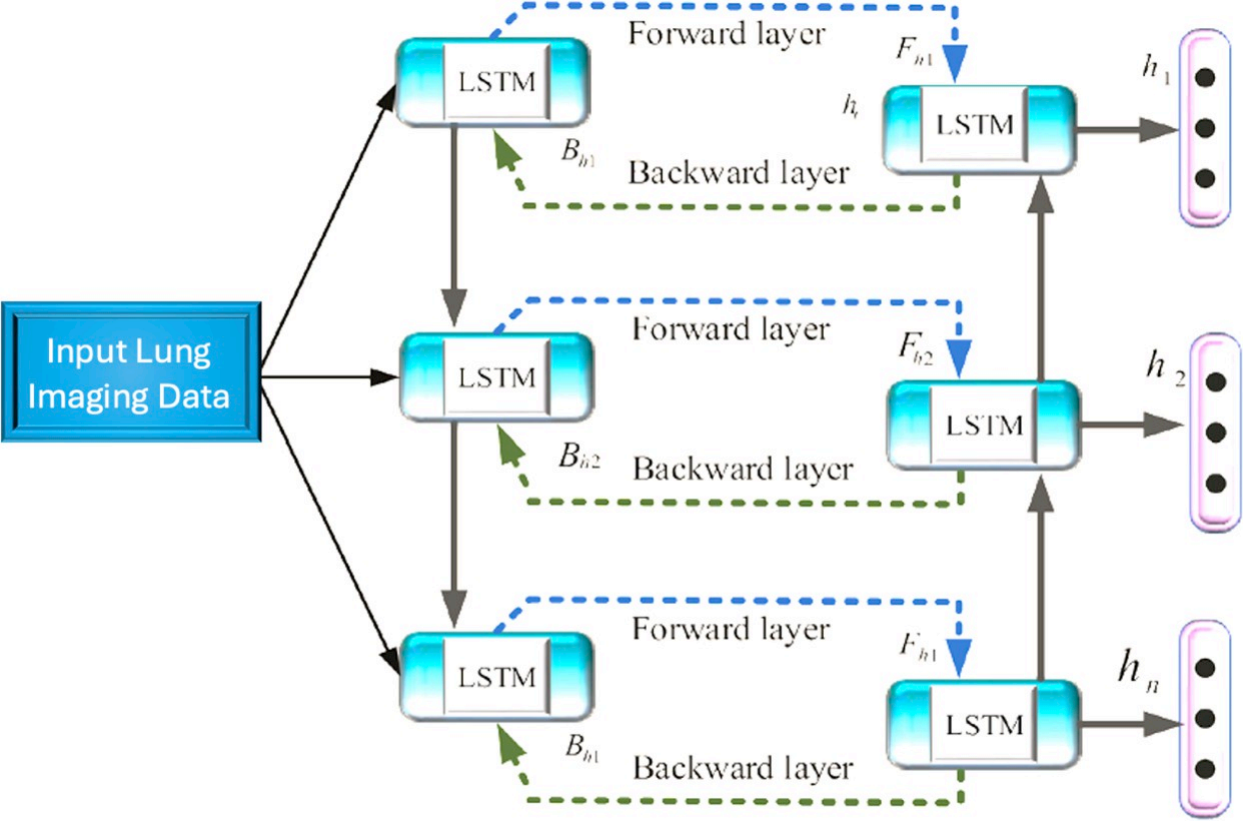
Bi-LSTM architecture.

In our proposed model, we employ a Bidirectional LSTM (Bi-LSTM) as reflected in Fig 2 that combines the information from both forward and backward layers. For each time step, the forward LSTM layer calculates the hidden vector Fht based on the previous hidden vector Fht-1 and the input k-space data *K*_*d*_. Similarly, the backward LSTM layer calculates the hidden vector Bht based on the opposite previous hidden vector *Bh*_*t*-1_ and the input k-space data *K*_*d*_. Finally, the forward hidden vector *Fh*_*t*_ and the backward hidden vector *Bh*_*t*_ are fused to obtain the final hidden vector for the Bi-LSTM model. The backward and forward hidden layer vectors are represented as *Bh*_1_, *Bh*_2_, *Bh*_3_, ………, *Bh*_*n*_ and *Fh*_1_, *Fh*_2_, *Fh*_3_, ……*Fh*_*n*_, respectively. The final hidden vector of the Bi-LSTM model is given by Eq (11).


$$h_{t}=\left\lceil F_{ht},B_{ht}\right\rceil$$ (11)

### 3.6. Bayesian Optimization (BO)

To optimize the hyperparameters of the Random Forest (RF) and Artificial Neural Network (ANN) models for accurate solar still hourly production forecasting, Bayesian Optimization Algorithm (BOA) was employed. BOA is a powerful technique for finding the global optimum of a black-box function, especially when function evaluations are computationally expensive [10, 64–66].

In this study, the 5-fold cross-validation accuracy was used as the objective function for BOA to prevent overfitting. The input variables (X) of BOA were the hyperparameters of the RF and ANN models, including the number of trees, maximum tree depth, learning rate, and number of neurons. The output variable (y) was the accuracy of the 5-fold cross-validation. The Upper Confidence Bound (UCB) acquisition function was selected for BOA, as it effectively balances exploration and exploitation. The BOA algorithm proceeds as follows:

1. In equation (VΙ), the Gaussian Procedure Model *GM* is built based on the collected data D.
GM~N(0,C) (12)
  Where *C* is the Kernel Matrix
$$C=\left[\begin{array}{ccc} C\left(y_{1},y_{2}\right) & \cdots & C\left(y_{1},y_{t}\right)\\ \vdots & \cdots & \vdots \\ C\left(y_{t},y_{1}\right) & \cdots & C\left(y_{t},y_{t}\right) \end{array}\right]$$ (13)
$$C\left(y_{j},y_{k}\right)=exp\left(-\frac{1}{2}{\left\|y_{j}-y_{k}\right\|}^{2}\right)$$ (14)
2. In the second step of the process, the next position (*y*_*t*+1_) to sample is chosen by the acquisition function. This is the place at which the measured feature (GM) is anticipated to be the best (an accuracy that is achieved by fivefold confirmation.). After that, the Gaussian process model (GM) undergoes modification by incorporating the most recent assessment (*y*_*t*+1_) into its framework.
3. The two processes are continued until the state that is considered to be terminal is attained.

Bayesian optimization is a technique used to find the global maximum of an unknown function. It is particularly useful when function evaluations are expensive, such as in our case where each evaluation involves conducting experiments on a physical system. Bayesian optimization models the objective function as a Gaussian Process (GP). By utilizing the posterior mean and variance of the GP, it can efficiently select the next evaluation point. The GP-Upper Confidence Bound (GP-UCB) acquisition function is a commonly used method for selecting the next sample location. This method balances exploration (trying new regions) and exploitation (focusing on promising areas) to efficiently optimize the function.


$${\text{b}}_{n}=\underset{\text{b}\in \text{B}}{\mathrm{ArgMax}}\;U_{n-1}(\text{b})+B_{n}^{\frac{1}{2}}\delta_{n-1}(\text{b}),$$ (15)

Where ℬ*_n_* is a scalar that varies with each iteration and indicates the level of confidence for the Gaussian Procedure. The Gaussian Process (GP) method selects the next evaluation point based on the uncertainty of the predicted function value. Specifically, it chooses points where the confidence interval of the GP’s prediction is the widest. By iteratively evaluating the function at these points, the GP refines its prediction and reduces uncertainty, leading to a more accurate estimation of the global maximum. While the GP-UCB method can be computationally expensive, it’s important to note that computational resources are relatively inexpensive. However, Bayesian optimization is most effective for problems with a limited number of parameters. To address this limitation, techniques like dimensionality reduction or incorporating additional structures into the kernel function can be employed to scale the method to larger problems.

### 3.7. Optimized Bi-LSTM model algorithmic steps

The following algorithmic steps are performed using Bi-LSTM model:

1 def objective_function(hyperparameters):
      Create a Bi-LSTM model with the given hyperparameters
    model = create_lstm_model(hyperparameters)
    ii) Compile the model with regularization
    model.compile(optimizer = hyperparameters[’optimizer’],
      loss = hyperparameters[’loss’],
      metrics = [’accuracy’])
    iii) Train the model with data augmentation
    for epoch in range(hyperparameters[’epochs’]):
     for batch in data_generator:
      X_batch, y_batch = batch
      X_augmented = augment_data(X_batch)
      model.train_on_batch(X_augmented, y_batch)
    iv) Evaluate the model on the validation set
    validation_acc = model.evaluate(X_val, y_val)[1]
    return -validation_acc # Maximize validation accuracy
2 Define the hyperparameter space of Bi-LSTM
    hyperparameters = {
     ’learning_rate’: (1e-4, 1e-2),
     ’batch_size’: (16, 128),
     ’units’: (32, 128),
     ’dropout’: (0.1, 0.5),
     ’optimizer’: [’adam’, ’rmsprop’, ’sgd’],
     ’loss’: [’binary_crossentropy’, ’categorical_crossentropy’],
     ’epochs’: (10, 100)
3 Create a Gaussian Process Regressor
    kernel = Matern(length_scale = 1.0, nu = 1.5)
    gpr = GaussianProcessRegressor(kernel = kernel)
4 Initialize the Bayesian optimization process
    X_init = np.random.uniform(low = 0.0, high = 1.0, size = (10, len(hyperparameters)))
    y_init = np.array([objective_function(hyperparameters) for hyperparameters in X_init])
    gpr.fit(X_init, y_init)
5 Perform Bayesian optimization
    num_iterations = 50
    for i in range(num_iterations):
     i)Sample a new hyperparameter configuration
     x_next = gpr.sample_optimization_target(n_samples = 1)[0]
     ii) Evaluate the objective function at the new configuration
     y_next = objective_function(x_next)
     iii) Update the Gaussian Process regressor
     gpr.fit(np.vstack([X_init, x_next]), np.hstack([y_init, y_next]))
     iv) Update the best hyperparameters
     best_hyperparameters = x_next if y_next < best_y else best_hyperparameters
    return best_hyperparameters

### 3.8. Performance evaluations measures

To assess the performance of our proposed system, we employed standard machine learning classification metrics: precision (positive predictive value), negative predictive value, specificity, sensitivity, and overall accuracy [67]. These metrics evaluate the system’s ability to correctly identify lung cancer cases.

### 3.9. Confusion matrix

This research employs a confusion matrix, a commonly used tabular format for assessing the performance of classification models on a given test dataset. The test data consists of known true positive and true negative values. Although the confusion matrix concept is relatively simple, the associated terminology can be misleading.

A confusion matrix is a visualization tool used in machine learning, particularly in classification problems, to evaluate the performance of a model. It provides a tabular representation of the predicted and actual classes. Here are the key parameters and their definitions:

True Positive (TP): Correctly predicted positive instances.
True Negative (TN): Correctly predicted negative instances.
False Positive (FP): Incorrectly predicted positive instances (type I error).
False Negative (FN):Incorrectly predicted negative instances (type II error).

The confusion matrix offers a detailed assessment of our model’s performance by categorizing its predictions. Table 1 presents the confusion matrix, which calculates various performance metrics based on actual and predicted values.

**Table 1:** Lung cancer detection based by utilizing optimized machine learning models and extracting hand-crafted GLCM features using 10-fold CV.

Method	Sensitivity	Specificity	PPV	NPV	Accuracy	F1-Score	AUC
SVM Linear	1.0000	0.9199	0.8620	1.0000	0.9466	0.9259	0.9946
SVM Quadratic	0.9971	0.9877	0.9803	0.9982	0.9913	0.9858	0.9998
SVM Cubic	0.9972	1.0000	1.0000	0.9982	0.9989	0.9958	0.9994

PPV: Positive predictive value, NPV: negative predictive value, AUC: area under the receiver operating characteristic curve

#### 3.9.1. Sensitivity

True positive rate (TPR) or sensitivity quantifies a classifier’s ability to correctly identify positive cases. Essentially, it measures the probability that a diseased individual will be accurately diagnosed as such. The following equation calculates sensitivity:

$$Sensitivity=\frac{TP}{TP+FN}$$ (16)

This indicates the likelihood of a correctly positive test result for a patient diagnosed with the disease.

#### 3.9.2. Specificity

True negative rate (TNR) or specificity complements sensitivity by assessing the model’s ability to correctly identify negative instances. It measures the proportion of actual negative cases that are accurately classified as negative. The following equation calculates specificity:

$$Specificity=\frac{TN}{TN+FP}$$ (17)

This indicates the likelihood of a correctly negative test result for a truly healthy patient.

#### 3.9.3. Positive Predictive Value (PPV)

Precision, PPV, measures a classifier’s ability to correctly predict positive instances. Mathematically, PPV can be calculated as:

$$PPV=\frac{TP}{TP+FP}$$ (18)

#### 3.9.4. Negative Predictive Value (NPV)

The NPV also known as negative precision, assesses a classifier’s ability to correctly predict negative instances. Mathematically, NPV can be calculated using the following equation:

$$NPV=\frac{TN}{TN+FN}$$ (19)

#### 3.9.5. Accuracy

Accuracy is a common metric for evaluating a classification model’s overall performance. It represents the proportion of correct predictions among all predictions. Mathematically:

$$Accuracy=\frac{TP+TN}{TP+FP+FN+TN}$$ (20)

### 3.10. Training and testing data formulation

To evaluate and optimize classifier performance, this study employed 10-fold Jackknife cross-validation, a robust technique suitable for limited datasets. This method involves partitioning the data into ten equal folds. Each fold is sequentially used for testing, while the remaining nine folds serve as training data. This process is repeated ten times, ensuring comprehensive evaluation of model performance.

Cross-validation is a technique used to assess the performance of a machine learning model. In this approach, the dataset is divided into multiple subsets. A portion of the data is used to train the model, while the remaining portion is used to evaluate its performance. A common example is 10-fold cross-validation. Here, the dataset is split into ten equal-sized folds. Nine folds are used for training, and the 10th fold is used for validation. This process is repeated ten times, with each fold serving as the validation set once. The final performance metric is the average of the ten iterations. 10-fold cross-validation offers a more reliable estimate of model performance compared to a single train-test split. It allows the model to be evaluated on a larger portion of the dataset while remaining computationally efficient.

**Fig 3:**
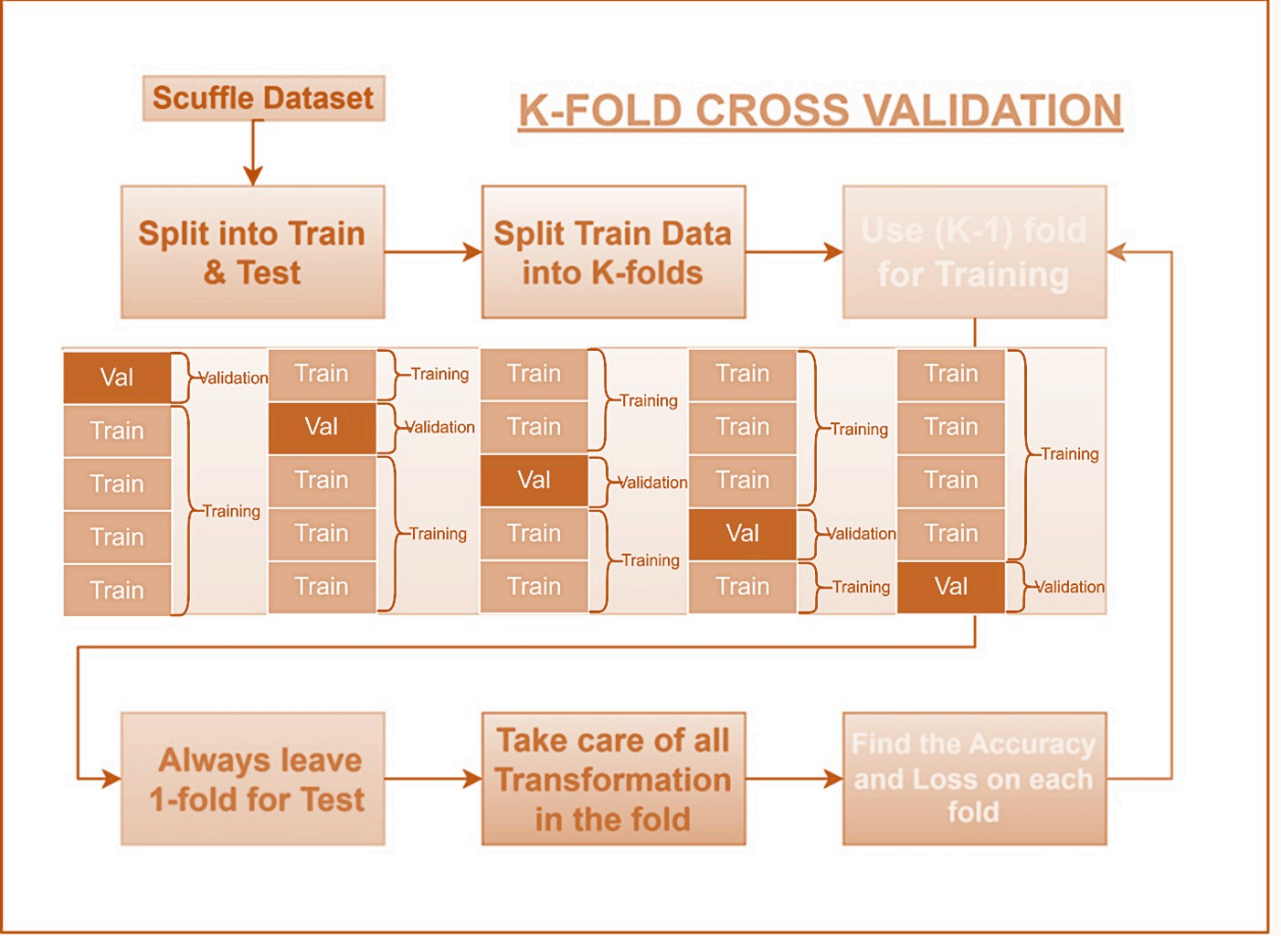
K-fold cross validation method for training/ testing data formulation.

Fig 3 illustrates the 10-fold cross-validation process, a common technique in machine learning to assess model performance. This process involves the following steps:

1. Data Division: The dataset is divided into ten equal-sized folds.
2. Model Training and Evaluation: Nine folds are used to train the model, while the remaining fold is used for validation.
3. Iteration: This process is repeated ten times, with each fold serving as the validation set once.
4. Performance Averaging: The performance metrics from each iteration are averaged to obtain a final estimate.

By training and evaluating the model multiple times, 10-fold cross-validation provides a more reliable assessment of its performance compared to a single train-test split. This is particularly beneficial when dealing with smaller datasets, as it maximizes the utilization of available data for both training and validation.

Given Data:

- NSCLC: 377 images
- SCLC: 563 images

Total Dataset: 377 + 563 = 940 images

This technique involves dividing the dataset into 10 equally sized folds. In each iteration:

1. Training: 9 folds (approximately 846 images) are used to train the model.
2. Testing: 1 fold (approximately 94 images) is used to evaluate the model’s performance.

This process is repeated 10 times, with each fold serving as the testing set once. The final performance metric is the average of the 10 iterations.

## 4. Results and discussions

This study aimed to enhance lung cancer detection through a dual approach. The first approach involved extracting hand-crafted features, including GLCM to capture distinct image characteristics. These features were subsequently fed into robust machine learning algorithms. The second approach explored the potential of deep learning by employing LSTM algorithms.

**Fig 4:**
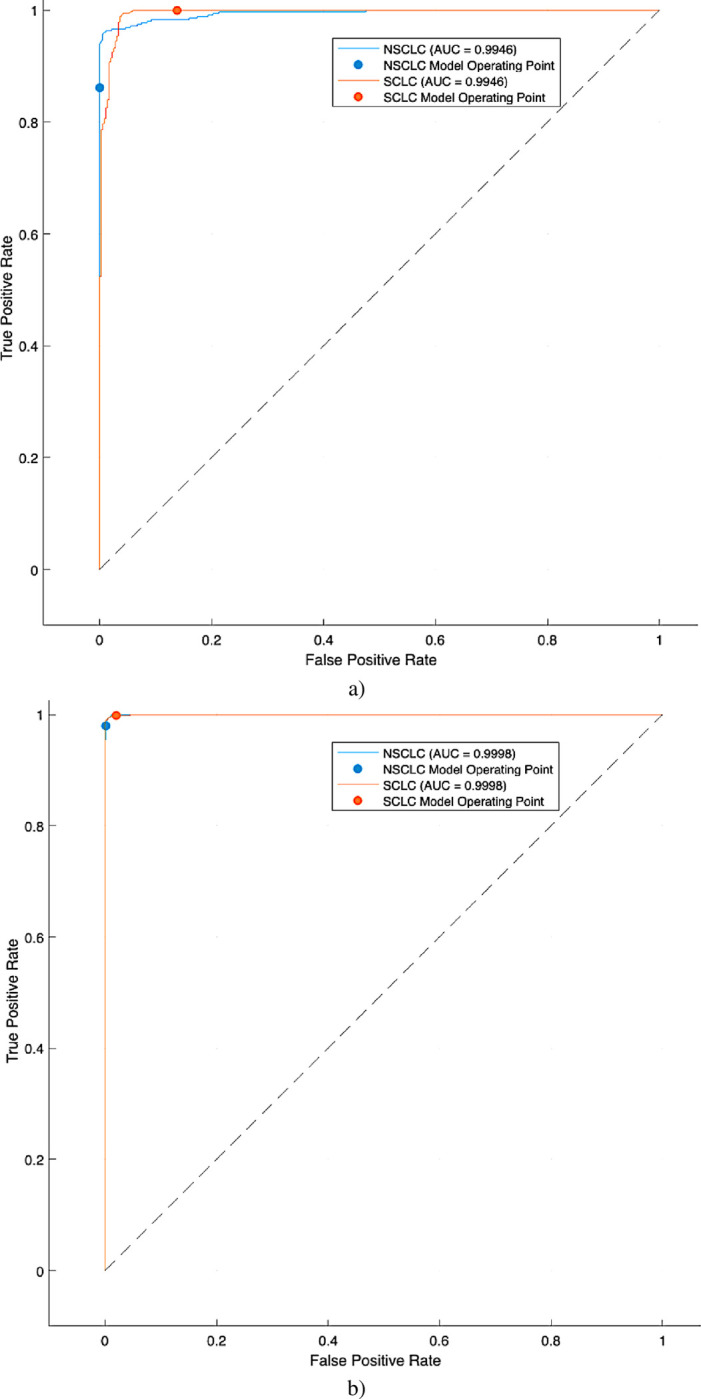
AUC to distinguish SCLC from NSCLC utilizing different machine learning and deep learning algorithms a) SVM Linear, b) SVM Quadratic with 10 fold cross validation (CV).

The Table 1 and Fig 4 presents the lung cancer detection performance using ML SVM kernels with 10-fold cross validation. The SVM Cubic model demonstrated the highest accuracy and F1-score, making it the most effective classifier for lung cancer detection in this study. It correctly identified 99.72% of true lung cancer cases and 100% of true non-cancer cases. Additionally, it had a perfect positive predictive value, meaning all positive predictions were accurate. While the SVM Quadratic model also performed exceptionally well, with high sensitivity, specificity, and F1-score, the SVM Cubic model’s slightly superior performance in terms of accuracy and F1-score suggests it is the most reliable choice for this task.

**Fig 5:**
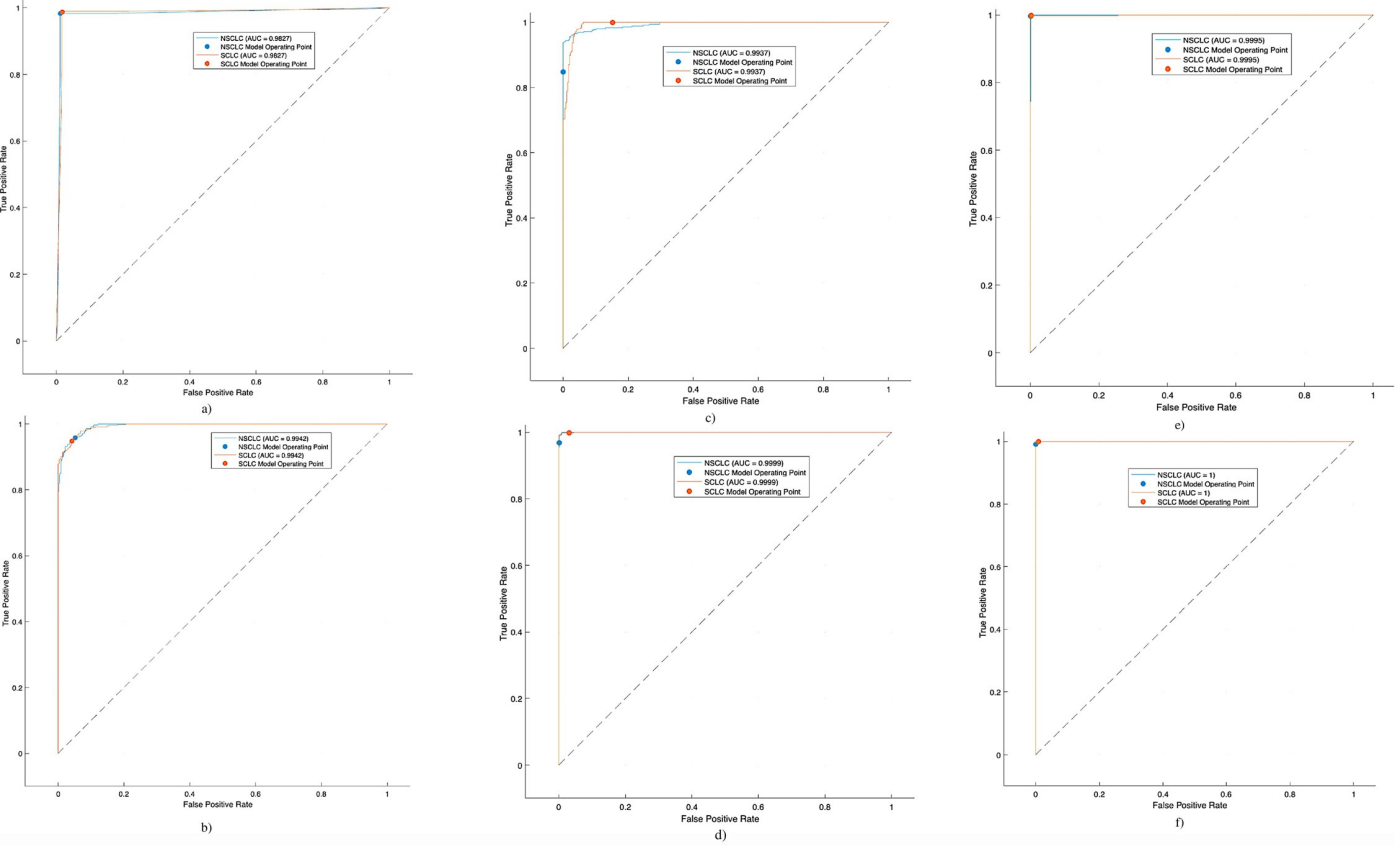
AUC to distinguish SCLC from NSCLC utilizing different machine learning and deep learning algorithms a) Decision Tree, b) Naïve Bayes, c) SVM Linear, d) SVM Quadratic, e) SVM Cubic, f) LSTM with 5 folds CV.

The Table 2 and Fig 5 shows the performance of various machine learning algorithms in detecting lung cancer using hand-crafted features extracted from GLCM with 5-fold cross-validation. The SVM Cubic consistently outperforms other models, achieving the highest scores in most metrics. It excels in sensitivity (99.72%), specificity (99.82%), PPV (99.72%), NPV (99.82%), accuracy (99.78%), F1-Score (99.44%),and AUC (99.95%). SVM Linear has the highest sensitivity (100%), but its lower specificity and PPV limit its overall performance. Decision Tree and SVM Quadratic both demonstrate excellent specificity and AUC, but their sensitivity and F1-Score are slightly lower than SVM Cubic. Naïve Bayes performs the worst among the models, with lower scores across most metrics. For lung cancer detection based on hand-crafted GLCM features, SVM Cubic is the most recommended model due to its superior performance in all key metrics. It provides a reliable and accurate solution for this task. However, the choice of the best model may depend on specific application requirements and the relative importance of different metrics.

**Table 2:** Lung cancer detection based by utilizing optimized machine learning models and extracting hand-crafted GLCM features using 5-fold CV.

Method	Sensitivity	Specificity	PPV	NPV	Accuracy	F1-Score	AUC
Naïve Bayes	0.9214	0.9727	0.9577	0.9485	0.9521	0.8654	0.9942
Decision Tree	0.9803	0.9893	0.9831	0.9876	0.9858	0.9624	0.9827
SVM Linear	1.0000	0.9125	0.8479	1.0000	0.9412	0.9177	0.9937
SVM Quadratic	0.9971	0.9808	0.9690	0.9982	0.9869	0.9800	0.9995
SVM Cubic	0.9972	0.9982	0.9972	0.9982	0.9978	0.9944	0.9995

PPV: Positive predictive value, NPV: negative predictive value, AUC: area under the receiver operating characteristic curve

The Table 3 presents the computational performance of various machine learning and deep learning algorithms. The comparative analysis of various machine learning models for lung cancer prediction reveals that SVM Cubic consistently outperforms other models in terms of accuracy, achieving a rate of 99.80%. While LSTM also demonstrates high accuracy (99.87%), its significantly longer training time and higher computational complexity may limit its practicality in certain applications. Naïve Bayes, on the other hand, offers a good balance between accuracy (95.20%) and computational efficiency, making it suitable for large datasets. SVM Linear and SVM Quadratic exhibit moderate accuracy and computational complexity, while Tree strikes a balance between the two with reasonable accuracy (98.60%) and low computational cost. Ultimately, the choice of the best model depends on the specific requirements of the application, such as the size of the dataset, the importance of accuracy, and the available computational resources.

**Table 3:** Comparative analysis of various performance metrics for predicting lung cancer.

Model Number (Features Selected/total)	Training time	Model size	Prediction speed	Accuracy % (Validation)	Total Cost (Validation)
Tree (22/22)	13.71 sec	8kB	2300 obs/sec	98.60	13
Naïve Bayes (22/22)	7.26 sec	335kB	51000 obs/sec	95.20	44
SVM Linear (22/22)	9.80 sec	46kB	1500 obs/sec	94.10	54
SVM Quadratic (22/22)	9.00 sec	17 kB	1500 obs/sec	98.70	12
SVM Cubic (22/22)	14.56 sec	15 kB	2000 obs/sec	99.80	2
LSTM (22/22)	1138.3 sec	722 kB	3500 obs/sec	99.87	129

The Table 3 provides insights into the computational efficiency of various machine learning models for lung cancer prediction. Tree-based models, like Decision Trees and Naïve Bayes, exhibit fast training times, small model sizes, and high prediction speeds. However, their accuracy can be lower compared to more complex models. SVM models, both linear and non-linear, offer moderate training times, relatively small model sizes, and reasonable prediction speeds. They generally achieve higher accuracy than simpler models but can be computationally more demanding, especially for non-linear kernels like quadratic and cubic. LSTM, a deep learning model, demands significantly more training time, a larger model size, and lower prediction speed compared to traditional machine learning models. However, it often achieves the highest accuracy, making it suitable for complex datasets and scenarios where accuracy is prioritized over computational efficiency. The choice of model depends on the specific requirements of the application, balancing accuracy, computational resources, and real-time constraints.

The Fig 5 presents the AUC to discriminate SCLC from NSCLC utilizing different machine learning and deep learning techniques. The highest AUC of 1.00 was obtained using LSTM followed by SVM Cubic with AUC of 0.9999.

**Fig 6:**
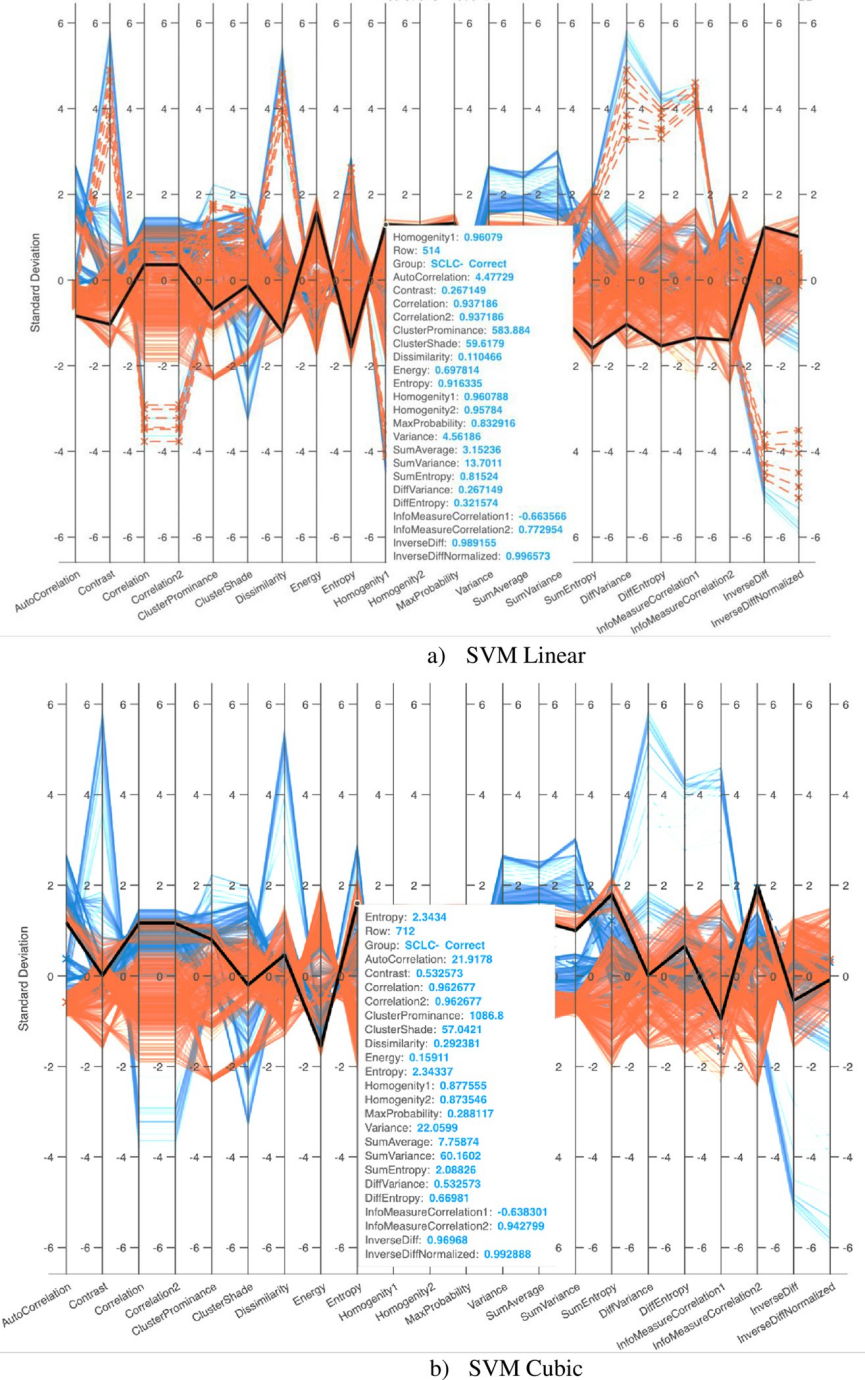
Parallel Coordinate graph to distinguish NSCLC from SCLC by computing GLCM features from lung cancer using a) SVM Linear, b) SVM Cubic.

Fig 6 illustrates parallel coordinate plots, a method for visualizing high-dimensional data. Each observation is depicted as a line connecting its values across various variables, revealing patterns and relationships within the data. The plots employ GLCM features extracted from MRI images of lung cancer types. The dataset comprises 356 NSCLC MRI subjects and 563 SCLC MRI subjects. Blue lines represent NSCLC cases, and red lines represent SCLC cases. Solid lines indicate correctly predicted cases, while lines with asterisks denote incorrectly detected cases.

In Fig 6A, SVM Linear achieves a validation accuracy of 98.69%, but with a higher incidence of incorrectly predicted data points, visualized as asterisks. Fig 6B demonstrates the predictions of SVM Cubic, which exhibits fewer incorrect classifications against each computed GLCM feature, also represented by asterisks.

The optimized Deep Learning LSTM model in Table 4 demonstrated exceptional performance in lung cancer detection. It achieved a perfect sensitivity of 100%, indicating that it correctly identified all actual lung cancer cases. Additionally, the model demonstrated a specificity of 99.82%, suggesting that it accurately identified 99.82% of true negative cases. The positive predictive value (PPV) of 99.72% indicates that 99.72% of positive predictions made by the model were indeed correct. Furthermore, the model achieved a perfect negative predictive value (NPV) of 100%, suggesting that all negative predictions made by the model were accurate. The overall accuracy of the model was 99.89%, which is exceptionally high and demonstrates its effectiveness in lung cancer detection. The F1-score of 99.86 reflects a good balance between precision and recall. Finally, the AUC of 1.0000 indicates that the model achieved an area under the ROC curve of 1,which is the maximum possible value and signifies perfect discrimination between lung cancer and non-lung cancer cases.

**Table 4:** Lung cancer detection using optimized deep learning LSTM with 10-fold cross-validation.

*Method*	*Sensitivity*	*Specificity*	*PPV*	*NPV*	*Accuracy*	*F1-score*	*AUC*
*Bi-LSTM*	1.0000	0.9982	0.9972	1.0000	0.9989	0.9986	1.0000

**Fig 7:**
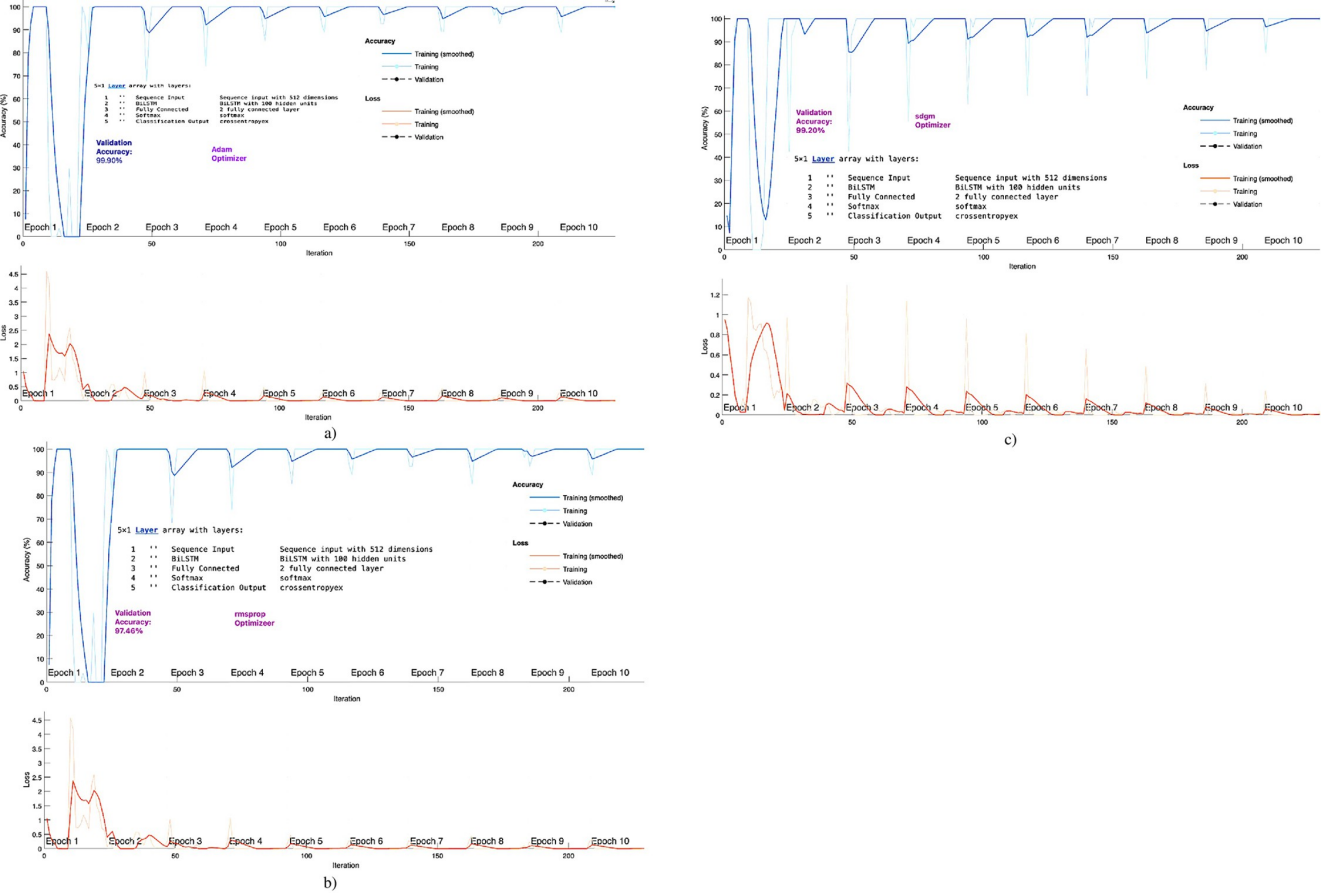
Accuracy-loss graph to detect Lung cancer using Bi-LSTM with different optimizers a) adam, rmsprop, c) sgdm.

The Fig 7 presents the accuracy-loss graph to distinguish the NSCLC from SCLC using optimized Bi-LSTM model with Bayesian optimization and using different activation functions. The adam optimizer yielded the highest validation accuracy of 99.90% followed by sgdm with validation accuracy 99.20% and rmsprop with validation accuracy of 97.46%.

The Table 5 demonstrates the significant performance improvement of the proposed methods, particularly the Bi-LSTM model, compared to previous studies. While prior research has relied on various feature engineering techniques, such as shape, intensity, texture, gradient, and morphology, the proposed approach leverages a combination of GLCM features and a deep learning architecture to capture intricate patterns within the data. This innovative approach has resulted in significantly higher sensitivity and accuracy in detecting lung cancer, positioning it as a promising technique for advancing the field of medical image analysis.

**Table 5:** Comparison of results with previous studies.

Author	Features Used	Performance
Teramoto et al. [68]	1. Shape	Sensitivity = 83.00%,
	2. Intensity	
Orozco et al. [69]	1. Texture	Sensitivity = 84.00%,
Guo et al. [70]	1. Texture	Sensitivity = 94.00%,
	2. Shape	
Messay et al. [71]	1. Shape	Sensitivity = 82.00%,
	2. Gradient	
	3. Intensity	
Retico et al. [72]	1. Texture	Sensitivity = 72.00%,
	2. Morphology	
**This study**	i) GLCM SVM Cubic	i) Sensitivity = 99.72%, Accuracy = 99.78%
	ii) Bi-LSTM	ii) Sensitivity = 100%, Accuracy = 99.89%

The Table 5 presents the comparative results with existing studies. In this study Teramoto et al. [68] computed the shape and intensity based features and achieved a sensitivity of 83%. Orozco et al. [69] computed texture features and achieved 84%. Guo et al. [70] calculated texture and shape features and obtained 94% of sensitivity. Retico et al. [72] utilized the texture and morphological features and yielded a sensitivity of 72%. This study utilized and optimized machine learning SVM cubic with GLCM features and obtained Sensitivity of 99.72% and accuracy of 99.78% and LSTM with Sensitivity of 100% and accuracy of 99.89%.

LSTMs excel at handling sequential data and capturing long-term dependencies. This makes them particularly suitable for medical image analysis tasks like lung cancer detection, where temporal patterns in image sequences are crucial. Unlike CNNs, which primarily focus on spatial patterns, LSTMs can effectively analyze the evolution of lung nodules or other abnormalities over time. Additionally, LSTMs can automatically learn relevant features from the input data, reducing the need for manual feature engineering. This makes them a powerful tool for improving the accuracy and reliability of lung cancer detection.

To enhance medical image analysis, high-quality images are essential. We initially improved image quality using robust enhancement techniques. Subsequently, we extracted relevant static features using machine learning algorithms. To optimize the performance of machine learning and deep learning LSTM models, we employed Bayesian optimization, a more powerful technique than random or grid search. Unlike previous studies that utilized fewer features and default parameters, we extracted a comprehensive set of robust GLCM-based features to capture more relevant information from lung cancer images. This enabled us to effectively distinguish between different image types. Our experimental results demonstrate that our proposed approach achieved superior detection performance compared to existing methods.

The SVM Cubic model demonstrated superior performance in terms of accuracy and F1-score, making it the most effective classifier for lung cancer detection. However, it’s important to consider the trade-off between accuracy and computational cost. While LSTM achieved the highest accuracy, its significantly higher training time and model size may limit its practical applicability. Future research should explore techniques to improve the computational efficiency of deep learning models while maintaining high accuracy. Additionally, incorporating more diverse datasets and advanced feature engineering techniques can further enhance the generalizability and robustness of these models. Ultimately, the goal is to develop accurate, efficient, and interpretable AI-driven tools to assist healthcare professionals in early diagnosis and treatment planning for lung cancer.

## 5. Conclusions

Lung cancer remains a major global health crisis, characterized by exceptionally low survival rates. As the most prevalent and deadliest cancer worldwide, its incidence has surged dramatically. This study aimed to differentiate between NSCLC and SCLC. To achieve this, we employed a combination of hand-crafted GLCM features and deep learning techniques, specifically the Bi-LSTM model. The exceptional performance of our Bi-LSTM model, as highlighted in the findings, underscores the potential of these methods for early lung cancer detection. Early detection is crucial for improving patient outcomes and reducing mortality rates.

### 5.1. Limitations and future directions

To further enhance the methodological rigor of our study, we plan to explore hybrid deep learning architectures and optimize model parameters. We will also expand our evaluation metrics and visualization techniques to provide a more comprehensive assessment of model performance. To address the limitations of our current dataset, we will test these methodologies on larger and more diverse lung cancer datasets. Additionally, we will incorporate clinical information alongside imaging features to improve diagnostic accuracy and predict disease recurrence, survival, and severity more effectively.

The current study employed a limited dataset and a restricted set of machine learning algorithms. Future work will involve exploring a wider range of algorithms and evaluating the clinical significance of the hybrid feature extraction approach. We will also compare our approach to existing clinical tools for lung cancer detection. To address these limitations, we will evaluate the hybrid feature extraction approach on larger and more diverse datasets, incorporating clinical information such as patient demographics, medical history, and symptoms. Furthermore, we will compare our approach to state-of-the-art lung cancer detection methods and explore its applicability to other medical imaging tasks, such as the detection of other cancers, neurodegenerative diseases, and cardiovascular diseases. By expanding the scope of our research, we aim to develop more robust and clinically relevant lung cancer detection models.

Finally, we will develop a hybrid feature extraction approach tailored to specific lung cancer subtypes, such as adenocarcinoma or squamous cell carcinoma, and utilize the proposed approach as a predictive model for lung cancer recurrence or survival.
